# Assessing Breast Cancer Margins Ex Vivo Using Aqueous Quantum-Dot-Molecular Probes

**DOI:** 10.1155/2012/861257

**Published:** 2012-12-24

**Authors:** Giang H. T. Au, Wan Y. Shih, Wei-Heng Shih, Linette Mejias, Vanlila K. Swami, Kimberly Wasko, Ari D. Brooks

**Affiliations:** ^1^School of Biomedical Engineering, Science and Health Systems, Drexel University, 3141 Chestnut Street, Philadelphia, PA 19104, USA; ^2^Department of Materials Science and Engineering, Drexel University, 3141 Chestnut Street, Philadelphia, PA 19104, USA; ^3^Department of Pathology and Laboratory Medicine, College of Medicine, Drexel University, 245 N 15th Street, Philadelphia, PA 19102, USA; ^4^Department of Surgery, College of Medicine, Drexel University, 245 N 15th Street, Philadelphia, PA 19102, USA

## Abstract

Positive margins have been a critical issue that hinders the success of breast- conserving surgery. The incidence of positive margins is estimated to range from 20% to as high as 60%. Currently, there is no effective intraoperative method for margin assessment. It would be desirable if there is a rapid and reliable breast cancer margin assessment tool in the operating room so that further surgery can be continued if necessary to reduce re-excision rate. In this study, we seek to develop a sensitive and specific molecular probe to help surgeons assess if the surgical margin is clean. The molecular probe consists of the unique aqueous quantum dots developed in our laboratory conjugated with antibodies specific to breast cancer markers such as Tn-antigen. Excised tumors from tumor-bearing nude mice were used to demonstrate the method. AQD-Tn mAb probe proved to be sensitive and specific to identify cancer area quantitatively without being affected by the heterogeneity of the tissue. The integrity of the surgical specimen was not affected by the AQD treatment. Furthermore, AQD-Tn mAb method could determine margin status within 30 minutes of tumor excision, indicating its potential as an accurate intraoperative margin assessment method.

## 1. Introduction

Breast cancer is one of the most common cancers among women in the United States and in Western countries. An estimated 226,870 cases of invasive breast cancer and 63,300 ductal carcinomas *in situ* (DCIS) will be diagnosed among women in the United States in 2012 [1]. Breast cancer is increasingly being diagnosed at an early stage [2] allowing treatment with breast conserving surgery (BCS), in which only the tumor and a small amount of surrounding normal tissue are removed.Multiple clinical trials have concluded that patients who undergo BCS with clean margin coupled with radiation have survival rates equivalent to those with mastectomy [3–6]. In addition, it was found that for every four local recurrences avoided in patients treated by BCS, one breast-cancer related death was averted [7]. Furthermore, morbidity and local recurrence rate are higher in patients with positive or close margin (16%) than those with negative margin (6%) [8, 9]. Positive and close margins usually refer to margins where cancer cells are present within 2 mm from the surface of the excised tissue. Consequently, it is best to have the tumor removed cleanly with negative margins on the first surgery [10]. 

Current BCS procedures rely on margin assessment in the pathology department to ensure completeness of tumor removal. It is only after the pathology report is completed that a final determination of surgical margin adequacy can be made. If the margin is found to be positive, reexcision is required, which often results in additional cost, let alone the additional pain to the patients. Currently, there is no real-time intraoperative method to rapidly and accurately assess the status of lumpectomy margins as a standard of care. Several techniques have been studied including gross examination, touch preparation cytology (TPC) [11, 12], frozen section analysis (FSA) [13, 14], radio-frequency spectroscopy (RFS) [15], tomography (TM) [2], and Raman spectroscopy (RS) [16, 17], each of which have various limitations with false negative diagnoses in 20–50% of the patients or prolong surgical time [18]. Although RFS, TM, and RS are more sensitive than TPC, they are limited by their dependence on tissue homogeneity. As a result, they are not as sensitive in heterogeneous tissues such as breast. It would be desirable to have a method that is not affected by tissue heterogeneity. 

On the other hand, molecular imaging has increasingly become more popular as a tool for fluorescence-guided surgery due to its sensitivity and specificity for cancer cells [19, 20]. Molecular imaging of cancer margin requires a biomarker that is specific to cancer but not the normal breast tissues. It also needs a fluorescent label that has little overlap with tissue autofluorescence. It is commonly accepted that there is no known unique biomarker for breast cancer due to the dynamic characteristics of the disease. However, for margin assessment purpose, the biomarker does not need to distinguish breast cancer from all other types of cancer but rather to distinguish cancer from the surrounding normal breast tissues. For this purpose, tumor-associated carbohydrate antigens (TACA) may be ideal as they are only associated with cancer but not the normal tissues. One of the most common TACAs is Tn antigen (GalNAc-*O*-Ser/Thr), a core glycan associated with mucins on the cancer cell surface of more than 90% of human epithelial carcinomas [21–24]. Tn antigen is formed due to the lack of activities of *β*1–3 D-galactosytransferase and *α*-2,6-sialyltransferase enzymes leading to incomplete elongation of O-glycan saccharide chains [25, 26]. It is a truncated form of a major type of glycosylation [27, 28]. Tn antigen is present in malignant breast lesions, invasive carcinomas[29, 30], and some types of benign lesions such as ducal hyperplasia or atypical lobular hyperplasia [30, 31]. Tn antigen has gained attention in antitumor vaccine applications as it is known to generate immune response in cancer patients. [32, 33]. From a study of Konska et al. [34], Tn antigen is expressed in 60%–80% of cancer cells in ductal carcinomas *in situ* (DCIS) and 20%–50% of cancer cells in lobular carcinoma *in situ*. In invasive ductal carcinoma (IDC), Tn is expressed in 70% of cancer cells of stage I cancer, 90%–100% of cancer cells in stage II cancer, and 40%–60% of cancer cells in stage III cancer. In addition, Tn is expressed in 20%–70% of cancer cells in invasive lobular carcinoma. The expression of Tn is uniform throughout the tumors [34].

The recent development of nanomaterials has provided considerable improvement in specificity and sensitivity for tumor imaging by using targeted contrasting agents [35, 36]. Quantum dots (QDs) are semiconductor nanoparticles that have unique photoluminescent capabilities. They exhibit a high fluorescence efficiency, are resistant to photobleaching [37], and comparable to green fluorescent protein (GFP) in size [38]. By changing particle's size, the emission spectra can be tunable which allows simultaneously imaging of different markers at the same pathological sites [39]. Bioimaging applications of QDs include cell labeling and tracking [40–42], cell proliferation [43], sentinel lymph node mapping [44], brain imaging [45], molecular beacons for DNA detection [46–48], and *in vivo* tumor detection [49, 50]. For specific target imaging, QDs can be coupled with antibody to detect biomarker on cell's surface. QDs can be used as labeling agents in immunofluroescence-based assay. 

Recent studies have shown that quantum dots can be directly made in an aqueous environment at room temperature (AQDs) with their capping ligands directly in place [51, 52]. The advantages of such AQDs are that they are more stable and easier to conjugate for bio-imaging. A recent conjugation study of CdSe AQDs showed that CdSe AQDs were more than 20 times more efficient in protein conjugation than commercial QDs which were made in an organic solvent (OQDs) and required ligand and solvent exchanges. Furthermore, CdSe AQDs are very bright with a high quantum yield (79%). It also worked well with a 700 nm long-pass emission filter [51], therefore will have little if any interference from tissue autofluorescence [53]. These attributes make CdSe AQDs a good candidate as fluorescent tag of molecular probes.

The purpose of this study is to demonstrate the use of a molecular probe consisting of a monoclonal antibody of Tn antigen coupled with CdSe AQDs to image margin status of human cancers grown in nude mice. This approach is molecularly specific, rapid, and not affected by tissue heterogeneity, which sets it apart from all other technologies that are available or being developed.

## 2. Materials and Methods

### 2.1. Cell Line and Cell Culture

The HT29 human colon cancer cell line was obtained from the American Type Culture Collection as it is the best characterized Tn antigen expressing in solid tumor that is easily available and reproducible. The HT29 cell line is a colorectal adenocarnioma which secretes carcinoembryonic antigen (CEA), transforming growth factor beta binding protein and mucin with high level of Tn anigen. Under standard growth conditions, the cells form a multilayer of non-polarized cells that display an undifferentiated phenotype [54]. Therefore, the HT-29 cells are aggressive. They form solid tumor in a short amount of time (2-3 weeks) compared to other carcinomas cell lines. HT 29 cells were maintained in McCoy's 5A medium supplemented with 10% fetal bovine serum (Bioexpress, Kaysville, UT) and 1% penicillin and streptomycin (Mediatech Inc., Manassas, VA) and cultured at 37°C in a 5% CO_2_ incubator. 

### 2.2. AQDs Synthesis and Conjugation

 CdSe AQDs were synthesized following the aqueous synthesis procedure developed by Li et al. [51, 55] with an optimal MPA : Cd : Se ratio = 4 : 3 : 1. The AQDs were conjugated to monoclonal Tn antigen antibody (mAb) (Tn218 IgM, Abcam, NJ) for direct tumor imaging. The details of the CdSe AQDs conjugation will be published in a separate publication. 

### 2.3. Subcutaneous Mouse Xenograft

Human HT29 cancer cells were harvested by trypsinizing a confluent T-150 cell culture flask. Viability was verified to be greater than 95% using trypan blue (Amresco, Solon, OH). The cells were resuspended at 10^6^ cells per 10 *μ*L of PBS, mixed 1 : 1 with Matrigel (BD Biosciences, San Jose, CA). The 10 *μ*L of prepared mixtures were injected subcutaneously in eight-week-old female nude mice having an average weight of 20 g. The tumors were allowed to grow for three weeks to reach the suitable size for study. 

### 2.4. Immunofluorescent Staining

To test the staining capability of the AQD-Tn mAb conjugate, HT29 cells were grown on cover glass overnight and then fixed with 4% paraformaldehyl for 15 minutes. Cells were washed with PBS three times. Cells were blocked with 10% normal goat serum for nonspecific binding for 1 hour at room temperature. Slides were then washed with 0.1% Tween/Tris buffer saline (TBS) for 3 times. AQD-Tn mAb complex was added and slides were incubated for 1 hour at room temperature. Slides were washed with TBS for 3 times and mounted with DAPI (Mounting medium with fluorescence, Vector Laboratories, CA, USA) for nucleus staining. Samples were stored in the dark at 4°C. A negative control was the sample without primary antibody. The slides were observed under an Olympus BX51 fluorescent microscope. 

### 2.5. Tumor Resection and Imaging

A total of 12 nude mice were used in the experiments. One mouse was used as a negative control without any cancer cell injection. Three weeks after injections, the mice were euthanized. Sharp dissection was used to excise the tumors with a small amount of the surrounding muscles still attached to the tumors. The tumors were round and regular in shape with unifocal characteristics on macroscopic appearance. The fresh tumors were immediately processed ex vivo with the staining procedure as described below, imaged and analyzed with IVIS imaging system (Lumina XR, Caliper, CA). First, the entire tumor's surface was washed with TBS then emerged in 1% bovine serum albumin solution (BSA) for nonspecific blocking for 10 minutes. Next, the tumor was removed from BSA solution and washed with TBS to remove BSA residue. The tumor was then immersed in AQD-Tn mAb solution for Tn-antigen staining of cancer cells. Finally, the tumor was washed again with TBS. The tumor was placed inside of IVIS for acquiring images. Each image acquisition would take about 30 seconds to complete. 

### 2.6. Optimal Blocking Time Evalutation

To find the optimal blocking time, fresh livers were washed with TBS for 2 min twice and then immersed in 1% bovine serum albumin (BSA) for various amounts of time. After washing in TBS three times, the livers were stained with AQD-Tn mAb complex for 1 hour at room temperature. The livers were suspended in 1% BSA solution by thin wire to maximize surface exposure. The blocking solution was stirred continuously to help the diffusion of BSA onto the tissue's surface. The livers were then washed again with TBS and imaged. After the optimal blocking time was identified, staining time was evaluated to develop the optimal margin assessing procedure for the whole tumor. 

### 2.7. Whole Mouse Imaging

One tumor was left inside a mouse. The mouse was euthanized and the entire peritoneum was opened to expose the tumor and internal organs on the ventral side. On the dorsal side, the tumor was left underneath the skin. However, since the skin around the shoulder blade was removed and exposed abdomen, the AQD-Tn mAb probe could get to the tumor even if it was covered with skin. Although the cancer cells were injected subcutaneously on the back of the mouse, the tumor invaded through the ventral side. There was little to no muscle on the tumor's surface. The whole animal was immersed in blocking solution and then AQD-Tn mAb probe suspensions. All the internal organs (such as lung, liver, kidney, etc.) and the tumor were exposed to the probe. This experiment was done to demonstrate the specificity and sensitivity of the probe when the tumor was surrounded by many normal tissues. The total staining procedure was 25 minutes. The mouse was then washed with TBS and imaged with the IVIS system. 

### 2.8. Interference on Microscopic Examination of the Operative Specimen

To examine the potential interference of the method with standard pathological procedures, we studied 10 tumor bearing nude mice. Each mouse had one control tumor and an average of two AQD-treated tumors. A total of 17 AQD-treated tumors and 10 control tumors were evaluated. The AQDs-treated tumors were stained with the AQDs-probe as described in Section 2.6. The control tumors were untreated by AQDs-probe. Both the AQDs-treated and the control tumors were submitted to the pathological department for the same regular pathological examination. Both the AQDs-treated tumors and the control tumors were fixed in 10% formaline for 12 hours and then embedded in paraffin. The blocks were cut into 5 *μ*m sections at the surfaces of the specimens where the AQDs-treated tumors were stained by the AQDs-probe. Both sets of sections (control and treated) were stained with H&E and immunohistochemistry (IHC) stained for 8 different markers: Tn antigen, VEGF, MSH6, MLH1, PMS2, p27, p53, and ki67. Interpretation was performed using Aperio ScanScope XT IHC Image Analysis algorithms (FDA-cleared *in-vitro* Diagnostic) and light microscopy (Olympus BX50) in the carcinoma component. 

## 3. Results 

### 3.1. Tn Antigen Expression in HT29 Cells

**Figure 1:**
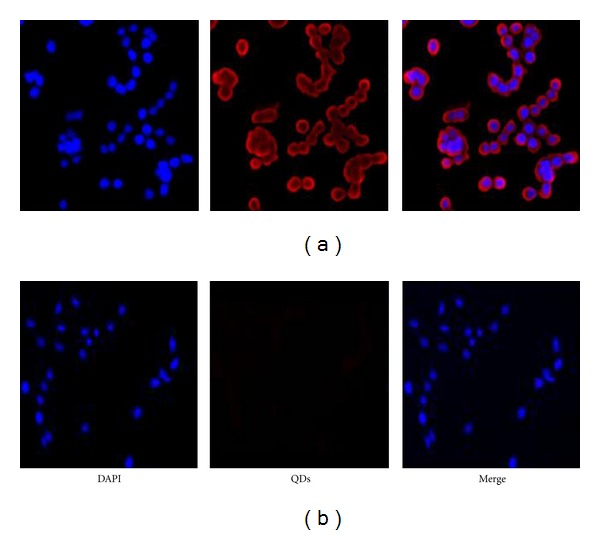
Immunofluroscent staining of HT29 cells for Tn antigen expression. (a) HT29 cells stained with AQD-Tn mAb complexes; (b) negative control, PEG activated AQD without antibody. Blue: nuclei, red: Tn antigen expression.

Immuofluorescent staining was performed on the HT29 cell lines to validate the functionality of QD-mAb complex. The expression of Tn antigen was evident as can be seen from Figure 1(a). AQDs without antibody showed no binding to HT29-cells (Figure 1(b)). These results indicate that QD-mAb complexes selectively bind to the Tn antigen protein. Furthermore, AQD-Tn mAb complex was compared with Cy3-labeled Tn mAb. Both AQD-Tn mAb and Cy3-Tn mAb bound to the HT29 cell at similar pattern, indicating AQD-Tn mAb complexes were functional (data not shown). 

### 3.2. Minimizing Background Signal

**Figure 2:**
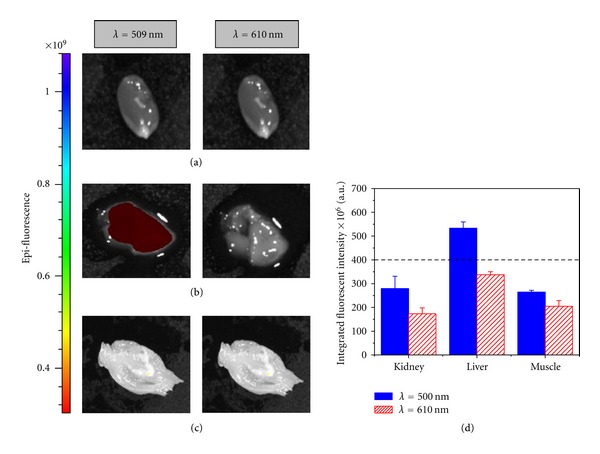
Background signal from normal tissues when excited at 460 nm. There were 2 emission cut-off wavelengths: 509 nm and 610 nm. (a) Kidney; (b) liver; (c) muscle; (d) integrated fluorescent intensity depended on the emission cut-off wavelength. Dash-line indicates background signal.

Autofluorescence has always been a challenge for fluorescent imaging especially in tissue with high adipose content such as breast and liver. Normal tissues are known to emit autofluorescent signal that ranges from 380 nm to 550 nm under UV light excitation (350–400 nm) [16, 53]. Here, we tried to establish a clear cut-off threshold to separate the background autofluorescence signal and the positive signal. Livers, muscles, and kidneys were used as normal tissue (negative controls) to evaluate the background threshold (Figure 2). At emission wavelength 509 nm, autofluorescent signal could be observed in liver (Figure 2(b)). Although other tissues did not show positive signal in the images, there were still background signals when the analysis was performed. With the emission at 610 nm, the background signals were reduced in all of the tissues especially livers (30% reduction) compared to emission at 509 nm (Figure 2(d)). A fluorescent intensity threshold of 400 × 10^6^ could be used as the cut-off to separate normal tissues since all the tissues autofluorescence background was below this level. 

### 3.3. Protocol Development

**Figure 3:**
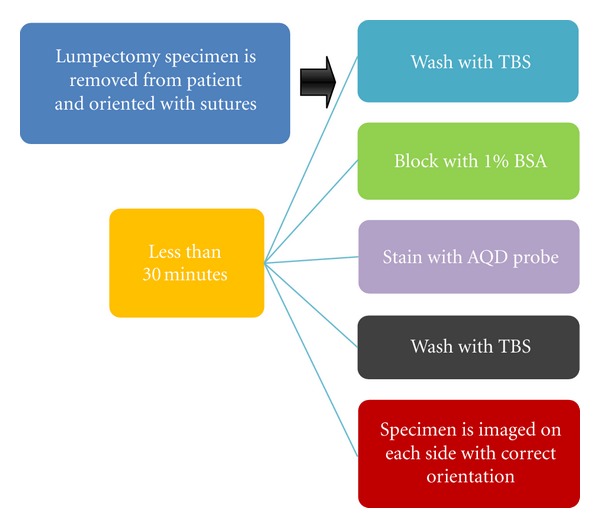
The proposed process of margin determination using AQD-Tn mAb probe.

**Figure 4:**
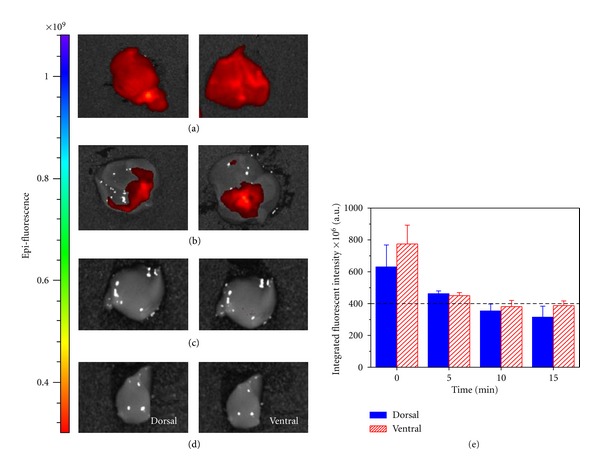
Blocking study for nonspecific staining using liver. (a) no blocking—0 min; (b) 5 min blocking; (c) 10 min blocking; (d) 15 min blocking; (e) quantification of the integrated fluorescent intensity versus time. Dash-line indicates acceptable background signal.

The total staining process of AQD-Tn mAb probe to evaluating excised tumor margin is summarized in Figure 3. First, lumpectomy specimen is removed from patient and oriented with sutures. The tumor is washed with TBS and then blocked with 1% BSA solution. Next, the tumor is removed from BSA solution, washed with TBS and immersed in AQD-Tn mAb probe suspensions for staining. Finally, the tumor is washed again with TBS and imaged on each side with correct orientation. There are two major steps that affect the sensitivity and specificity of AQD-Tn mAb probe: blocking time and staining time. First, blocking time was investigated. Different time periods were examined and it was narrowed down to 15 minutes as the sufficient blocking time. Smaller time intervals were studied to further shorten the blocking time. As shown in Figure 4(a), no BSA blocking resulted in strong nonspecific binding of the QD-mAb probe on liver's surface both dorsal and ventral sides. As the blocking time increased, nonspecific binding decreased and reached the saturated point at 10 minutes. The integrated intensity was similar between 10 minute and 15 minute blocking (Figures 4(c) and 4(d)). This was also at the same level as a control liver without QD-Tn mAb probe exposure (data not shown). Therefore, 10 minutes blocking should be sufficient to prevent nonspecific binding

### 3.4. Simulation of Intraoperative Margin Assessment

**Figure 5:**
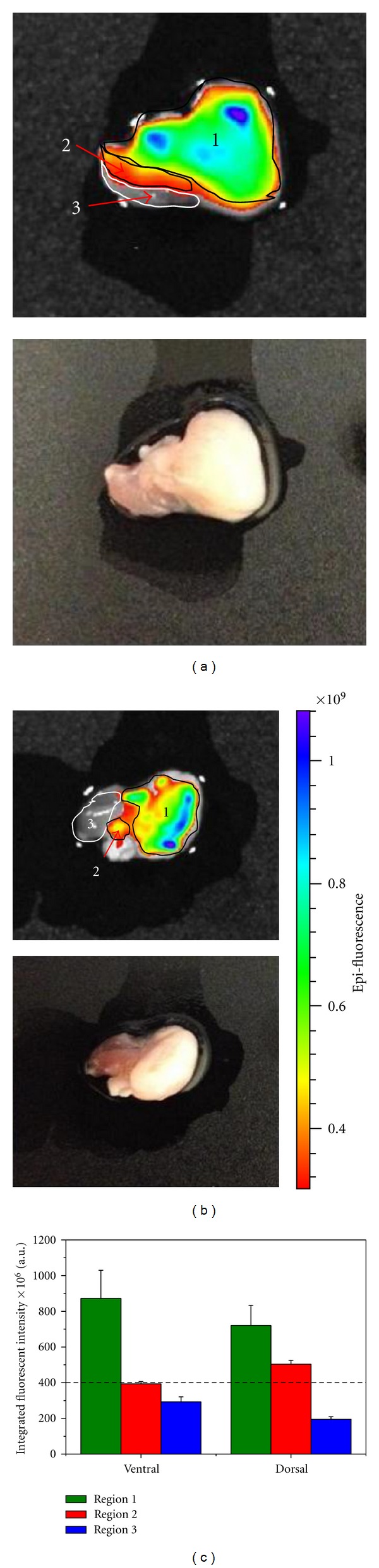
Animal tumor imaging. Top panel: fluorescent imaging using IVIS system. Bottom panel: bright field images of the same tumor. Two orientations of the tumor were imaged: (a) ventral side; (b) dorsal side. (c) The integrated fluorescent intensity was quantified using IVIS software for 3 regions of the tumor. Dash-line indicates the cut-off between normal and cancer areas.

As previously mentioned, although livers and kidneys showed no positive signal, they still had some background intensity around 400 × 10^6^. Thus, this was the cut-off level to differentiate between cancer and normal tissue. Optimal staining time was 15 minutes (data not shown). The excised tumors were divided into 3 different regions: a tumor region, a muscle region, and an overlap between tumor and muscle or margin region. Comparing the fluorescent image with the bright field image, we could see that bright dark to turquoise blue-region (region 1 in Figures 5(a) and 5(b)) correlated with the tumor and gray region correlated with the muscle (region 3). The tumors were clearly identified by the AQD-Tn mAb probe. AQD probe was also specific to the tumor and not the muscle as evidenced by the unstained muscle area. Region 2 was more ambiguous based on the image in Figures 5(a) and 5(b). In the dorsal view (Figure 5(a)), the color map showed that region 2 was red corresponding to integrated fluorescent intensity of less than 400 × 10^6^ (Figure 5(c)). This indicated the region to be free of cancer cells. Meanwhile, the ventral bright field image looked like muscle area whereas the fluorescent image indicated the presence of cancer cells with quantitative fluorescent intensity value of 503 × 10^6^, indicating the method was sensitive and specific to detect small non-palpable lesions. The red color in the images could be interpreted as negative region (integrated fluorescent intensity less than 400 × 10^6^).

**Figure 6:**
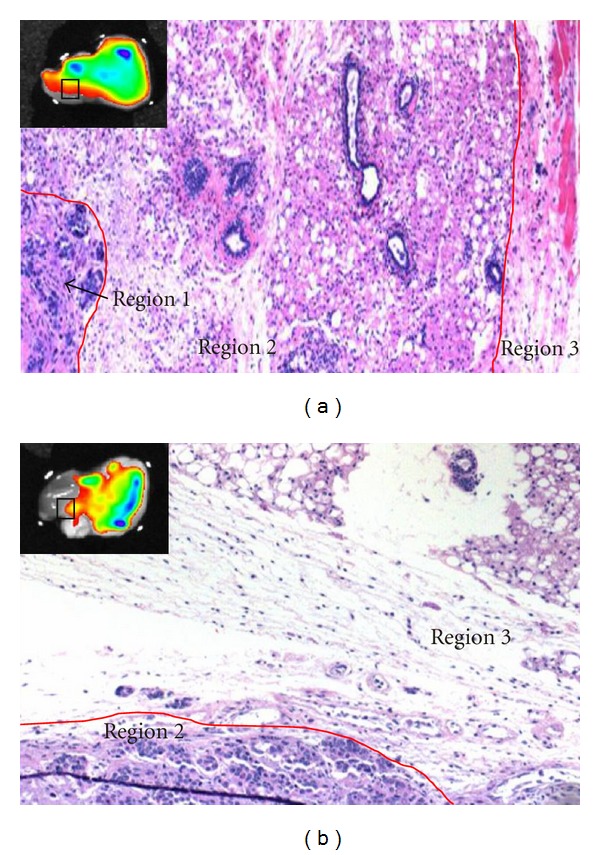
H&E stained sections correlated to the regions (square box) of the examined tumor. (a) Dorsal side; (b) Ventral side. Cancer cells were absent in region 2 of the dorsal side. Cancer cells were detected in region 2 of the ventral side by AQD-Tn mAb probe and confirmed by H&E stained section.

To further confirm the presence or absence of cancer cells in region 2 of both dorsal and ventral sides, the tumor was embedded in paraffin and examined using the H&E-stained sections of these regions.  Figure 6 showed the areas in the square boxes of the tumor, with each region separated by the red line. For the dorsal side, the square box contained all three regions—tumor, interface, and muscle. Meanwhile, the square box in the ventral side consisted of only region 2 and region 3 due to larger area of region 2 to be included in the image. Clearly, dorsal region 2 (Figure 6(a)) contained only inflammatory and fibroblasts cells, which correlated to the red color in the whole tumor examination indicating negative signal. The presence of cancer cells were observed in H&E section of ventral region 2 (Figure 6(b)), which confirmed the above positive assessment. The results suggested that this method was sensitive and specific to identify cancer cells in areas that could have been missed by gross examination during tumor removal process. By applying the quantitative analysis of AQD-Tn mAb probe signal, normal, and cancer regions could be distinguished in real-time. 

### 3.5. Whole Mouse Imaging

**Figure 7:**
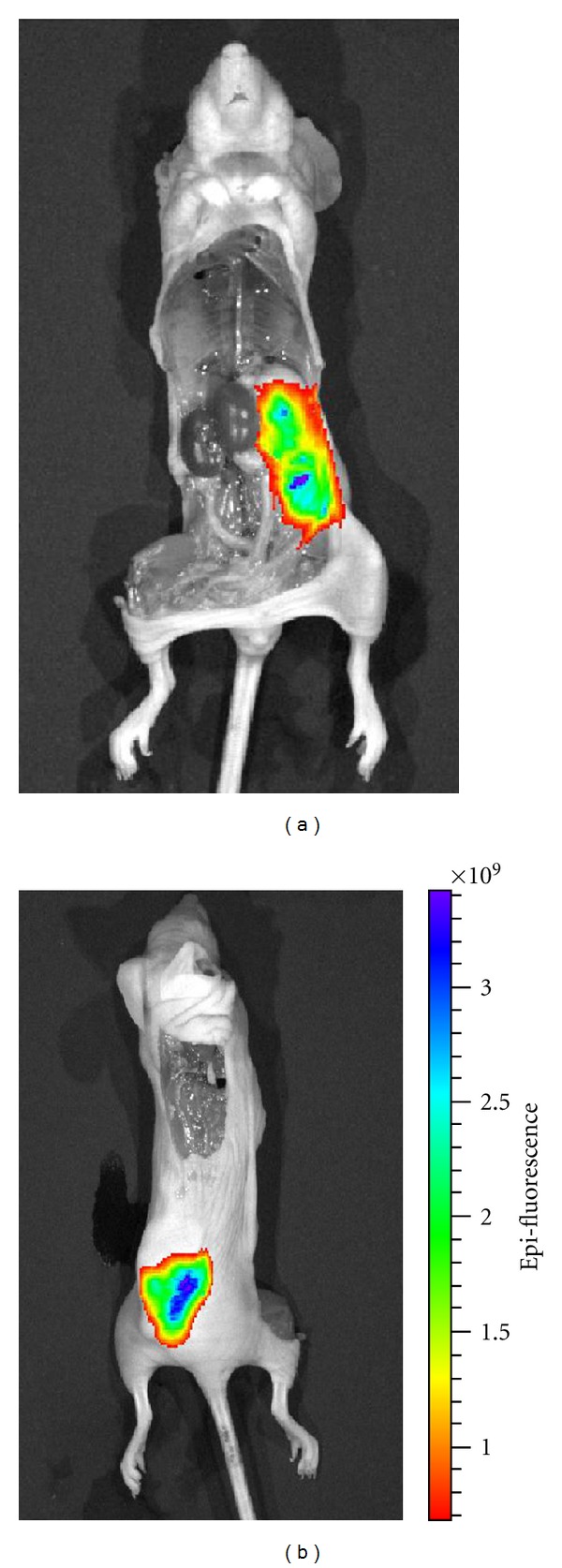
Whole mouse tumor imaging. (a) Ventral site where the tumor was exposed. (b) Dorsal site, tumor was underneath the skin. Other organs had negative signal, indicating AQD-Tn mAb prove was specific and sensitive to the tumor.

To further demonstrate the capability of the method, one tumor was left inside of the mouse body.  Figure 7 shows the dorsal and ventral pictures of the mouse. The tumor was exposed at the ventral side and underneath the skin at the dorsal side. We found that AQD-Tn mAb probe resulted in the ability to visualize areas of tumors that were not apparent with white light because the appearance of the tumor was not easily distinguished from the other tissues with good fluorescence contrast, indicating highly specific tumor targeting of AQD-Tn mAb probe. The positive signal was strong enough to identify the outline of the tumor surface (fluorescence was much greater than 400 × 10^6^). All the other organs showed no positive signal confirmed the specificity and sensitivity of the AQD-Tn mAb probe.

### 3.6. Interference on Microscopic Examination of the Operative Specimen

**Figure 8:**
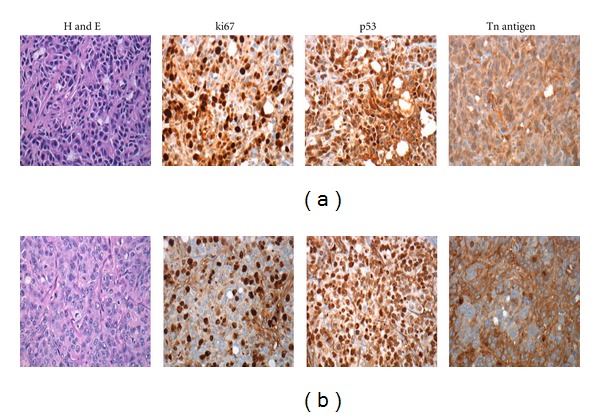
Interference examination of AQD-Tn mAb probe on standard pathological evaluations. (a) Control tumors; (b) AQD-treated tumors. Different markers were evaluated such as Tn antigen, p53, and ki67. No interference with the following pathological examination was found.

We examined the potential impact of AQD-Tn mAb probe on the microscopic examination of the operative specimen by submitting the treated tumors to pathology department for standard processing. Using multiple routine evaluated markers, we found that there was no difference between the H&E staining of an AQDs-stained tumor and that of a control tumor. There was also no difference between the IHC staining of the various markers: Tn antigen, VEGF, MSH6, MLH1, PMS2, p27, p53, and ki67 on an AQDs-treated tumor and that of the various markers on a control tumor. As examples, Figure 8 showed the H&E staining and IHC staining of ki67, p53, and Tn antigen of a control tumor (Figure 8(a)) and those of an AQD-treated tumor (Figure 8(b)). As can be seen, there was no difference between the H&E staining of the control tumor and that of an AQDs-treated tumor nor was there a difference between the IHC staining of ki67, p53, and Tn antigen of a control tumor and of an AQDs-strained tumor. Furthermore, quantitative grades of ki67 expression were 38.2 ± 5.2% in the control tumors and 31 ± 6.4% in AQD-treated tumors. For p53 expression, the quantitative grades were 38.6 ± 3% and 41.5 ± 3.7% in the control tumors and AQD-treated tumors respectively. This result clearly indicates that the AQDs-based assessment method would not interfere with the standard histological examinations of the surgical specimens.

## 4. Discussion 

It is well known that incomplete removal of a tumor is a major factor that compromises the long term survival rate of cancer patients. This study presents the first demonstration of molecular imaging for intraoperative ex vivo tumor margin assessment. By providing a quantitative threshold level, AQD-Tn mAb probe provides surgeons the ability to evaluate margin status in real-time, potentially reducing the number of positive margins found postoperatively, and thus reducing the need for the second operation and risk of local recurrence. AQD-Tn mAb effectively identified cancer areas that could be missed by the current gross visual examination. Furthermore, AQD-Tn mAb bound specifically to the cancer cells and not adipocytes and stromal cells as verified by histopathology.

Tissue autofluorescence is a serious background noise issue in any fluorescent imaging and can lead to false positives. Many biomolecules exhibits endogenous fluorescence including amino acids, structural proteins, and lipids. Their emission maxima range between 280 nm to 550 nm [53]. For epithelial tissues such as breast, the concentration of endogenous fluorophores can be substantial between the surface epithelium and the underlying stroma to result in strong autofluorescence in adipose tissue and the stroma. In this study, the CdSe AQDs were imaged with a 610 nm emission filter due to the constraint of the imaging system. However, the CdSe AQDs probe can be viewed with a 700 nm long-pass emission filter, which will allow the signal of the AQDs further separated from tissue autofluorescence, with a higher signal to noise ratio and make the QD-Tn mAb probe even more sensitive and specific in the future. Meanwhile, unlike fluorophores, AQDs can undergo constant light exposure with minimal photobleach, which often leads to loss of signal. AQDs allow convenience in handling the probe without the need of a dark room. 

Current technologies such as wire-guided localization (WGL) can perform intraoperative tumor localization with positive margin ranges from 23% to 46% [56, 57] and does not provide a clear three-dimensional image of tumor edges [58]. Ultrasound guided resection is limited to ultrasound visible tumors while specimen radiography detects clips or calcifications in a tumor specimen, but both are limited in ability to establish clear margins reliably [59]. New developing optical-based imaging technologies appear to be applicable for intraoperative imaging due to their portable size and low cost. For example, the optical spectroscopy method developed by Wilke et al. transforms optical images into tissue composition maps with parameters of total hemoglobin concentration, *β*-carotene concentration, and scattering [60]. The MarginProbe method [15] is a near-field radio frequency (RF) spectroscopy device that detects differences between dielectric properties of malignant and normal breast tissue. These methods, however, depend on the intrinsic measurements, such as tissue scattering and autofluorescence of the tissues, leading to unacceptable false-negative rates due to the high heterogeneity of malignant and benign tissues [15, 17, 61]. The present AQD-Tn mAb probe does not depend on the physical-mechanical characteristic of the tissue but assessing the differences between normal and cancer at the molecular level. TACA Tn antigen has been reported to be expressed exclusively in cancer cells and not normal tissue. Using this molecular signature of cancer cells, tissue heterogeneity is not an issue as the results presented above clearly showed that the AQD-Tn mAb probe was capable of displaying very small spots consisting of 100 to 200 cancer cells. This is a key advantage compared to most of the current developing optical-based imaging technologies which rely on signal average over a large area and thus are unable to image cancer in a heterogeneous background. 

Total margin evaluation time is one of the most critical requirements for intraoperative margin status determination. FSA has been reported to have good sensitivity and specificity to cancer cells but has difficulties in performing frozen sections on adipose tissue results in increasing surgery time and cost due to additional pathology evaluation [13]. The most significant disadvantage of FSA is the inability to evaluate the entire surface area with sampling rate of 10–15% surface area. Using antibody-antigen binding mechanism, the AQD-Tn mAb probe was able to stain and identify cancer areas quantitatively in less than 30 minutes to prevent the prolonged anesthesia period for patients. All sides of the tumor are evaluated which give the surgeon the exact location of cancer area. Furthermore, no additional intraoperative pathological evaluation is needed to decide whether an area contains cancer cells or not. Manipulations of the surgical specimen have no impact on the microscopic examination of the operative specimen as shown in the interference study is another advantage of this method. The specimen can undergo normal histologic examinations for further margin confirmation and other necessary markers evaluations.

In this study, we used HT29 colon cancer cell as our tumor model instead of a breast cancer cell line. HT29 cells expressed Tn antigen strongly without any need of transfection to express the protein, as verified by Western blot. Because this was a proof of concept study, we wanted to ensure that our tumors express the marker strongly so that we could control over the methodology development. Furthermore, we have showed that AQD-Tn mAb probe was capable of staining the whole tumor inside the mouse body locally once the tumor surface was exposed. Although there was positive signal of tumor underneath the skin at the dorsal side, the skin was relatively thin (less than 1 mm). With the depth of 2 mm (consider negative margin), no signal would be observed due to the penetration depth of 610 nm wavelength. We will examine this aspect in our future studies. The tumor imaging inside the mouse further indicated that AQD-Tn mAb probe was very sensitive and specific to cancer cells only. This can potentially be developed as a tool to examine the cavity after tumor is removed for additional information about the margin. 

## 5. Conclusion

In conclusion, we have demonstrated the use of a molecular probe AQD-Tn mAb to assess the surface of excised human cancers grown in nude mice. The AQD-Tn mAb molecular probe consisted of the antibody to target cancer-specific Tn-antigen on the cancer cell surface that is covalently linked to CdSe AQDs. The advantages of the CdSe AQDs as the fluorescent tag of a cancer molecular probe include brightness, without photo-bleaching, and can be accessible to 700 nm long-pass emission filter that minimizes background tissue autofluorescence. The results showed that the AQD-Tn mAb was effective to image tumor margin in less than 30 min. Tissue heterogeneity which was an issue for optical- and electrical-current-based imaging technologies did not have an effect in AQD-Tn mAb imaging due to its specific binding capability which allows a more precise margin assessment. The integrity of the surgical specimen was not affected by the AQD treatment and there was no difference in the quality and intensity of standard H&E as well as IHC stains. The AQD-Tn mAb molecular probe offers the potential to quantitatively and accurately assess margin during surgery to help reduce reexcision rate.
